# Extended-conjugation π-electron systems in carbon nanotubes

**DOI:** 10.1038/s41598-018-26379-4

**Published:** 2018-05-25

**Authors:** Kenshi Miyaura, Yasumitsu Miyata, Boanerges Thendie, Kazuhiro Yanagi, Ryo Kitaura, Yuta Yamamoto, Shigeo Arai, Hiromichi Kataura, Hisanori Shinohara

**Affiliations:** 1 Department of Chemistry & Institute for Advanced Research, Nagoya University, Nagoya, 464-8602 Japan; 2 Department of Physics, Tokyo Metropolitan University, Hachioji, 192-0397 Japan; 3 JST, PRESTO, Kawaguchi, Saitama 332-0012 Japan; 4 High Voltage Electron Microscope Laboratory, Institute of Materials and Systems for Sustainability, Nagoya University, Nagoya, 464-8602 Japan; 5 Nanomaterials Research Institute, National Institute of Advanced Industrial Science and Technology (AIST), Tsukuba, Ibaraki 305-8565 Japan

## Abstract

Extending π-electron systems are among the most important topics in physics, chemistry and materials science because they can result in functional materials with applications in electronics and optics. Conventional processes for π-electron extension, however, can generate products exhibiting chemical instability, poor solubility or disordered structures. Herein, we report a novel strategy for the synthesis of π-conjugated polymers within the interiors of carbon nanotubes (CNTs). In this process, thiophene-based oligomers are encapsulated within CNTs as precursors and are subsequently polymerized by thermal annealing. This polymerization increases the effective conjugation length of the thiophenes, as confirmed by transmission electron microscopy and absorption peak red shifts. This work also demonstrates that these polythiophenes can serve as effective markers for individual CNTs during Raman imaging with single-wavelength laser excitation due to their strong absorbance. In addition, stable carrier injection into the encapsulated polythiophenes is found to be possible via electrochemical doping. Such doping has the potential to produce π-electron-based one-dimensional conductive wires and highly stable electrochromic devices.

## Introduction

Because they represent cylindrical nanospaces with high thermal and chemical stability, the interiors of carbon nanotubes (CNTs) have been considered as potentially useful platforms for the fabrication of one-dimensional (1D) nanomaterials by molecular coalescence. One representative example is the formation of internal single-wall CNTs via the thermal coalescence of fullerene chains at high temperatures in the vicinity of 1000 °C^1^. In addition to several 1D inorganic materials constructed through filling^2,3^, the inner space of CNTs provides various molecular reactions that are difficult by conventional processes. Such molecular reactions have been applied to the synthesis of various 1D nanomaterials, such as graphene nanoribbons^4–11^, fullerene chains^12,13^, boron nitride nanotubes^14^, diamond nanowires^15,16^, and polyene chains^17,18^. Materials prepared within CNTs in this manner can be subsequently extracted from the solution phase^5,19^. The preparation of π-conjugated polymers in CNTs is also of particular interest, because of the remarkable electrical and optical properties of these polymers^20,21^.

**Figure 1:**
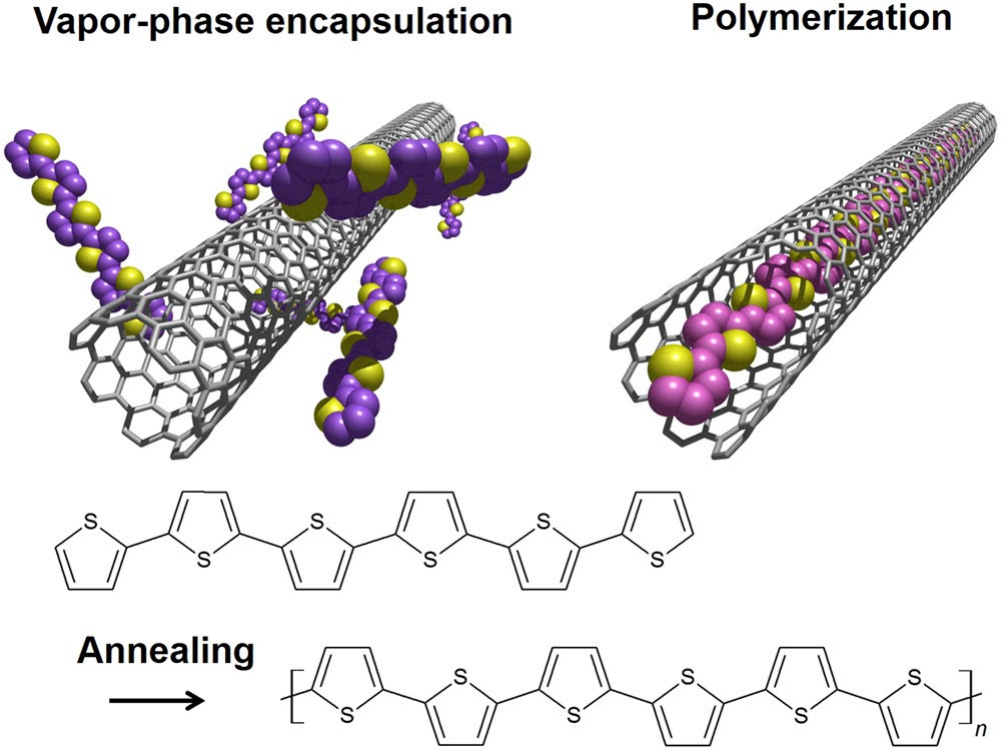
Strategy for synthesis of π-conjugated polymers in CNTs. Thiophene oligomers are encapsulated in a CNT via vapor phase process and are then polymerized by thermal annealing.

In the present work, the π-conjugated polymers shown in Fig. 1 were synthesized by self-assembly in the interiors of CNTs via vapor-phase encapsulation of thiophene-based oligomers, including α-quaterthiophene (4 T) and α-sexithiophene (6 T), which were subsequently polymerized by thermal treatment. These thiophene oligomers were selected because of their high thermal stability during encapsulation process and their strong optical absorption in the visible region^22^. After encapsulation, each sample was characterized using high-resolution transmission electron microscopy (HR-TEM) and optical absorption, Raman and photoluminescence (PL) spectroscopic analyses.

### Diameter dependent encapsulation

**Figure 2:**
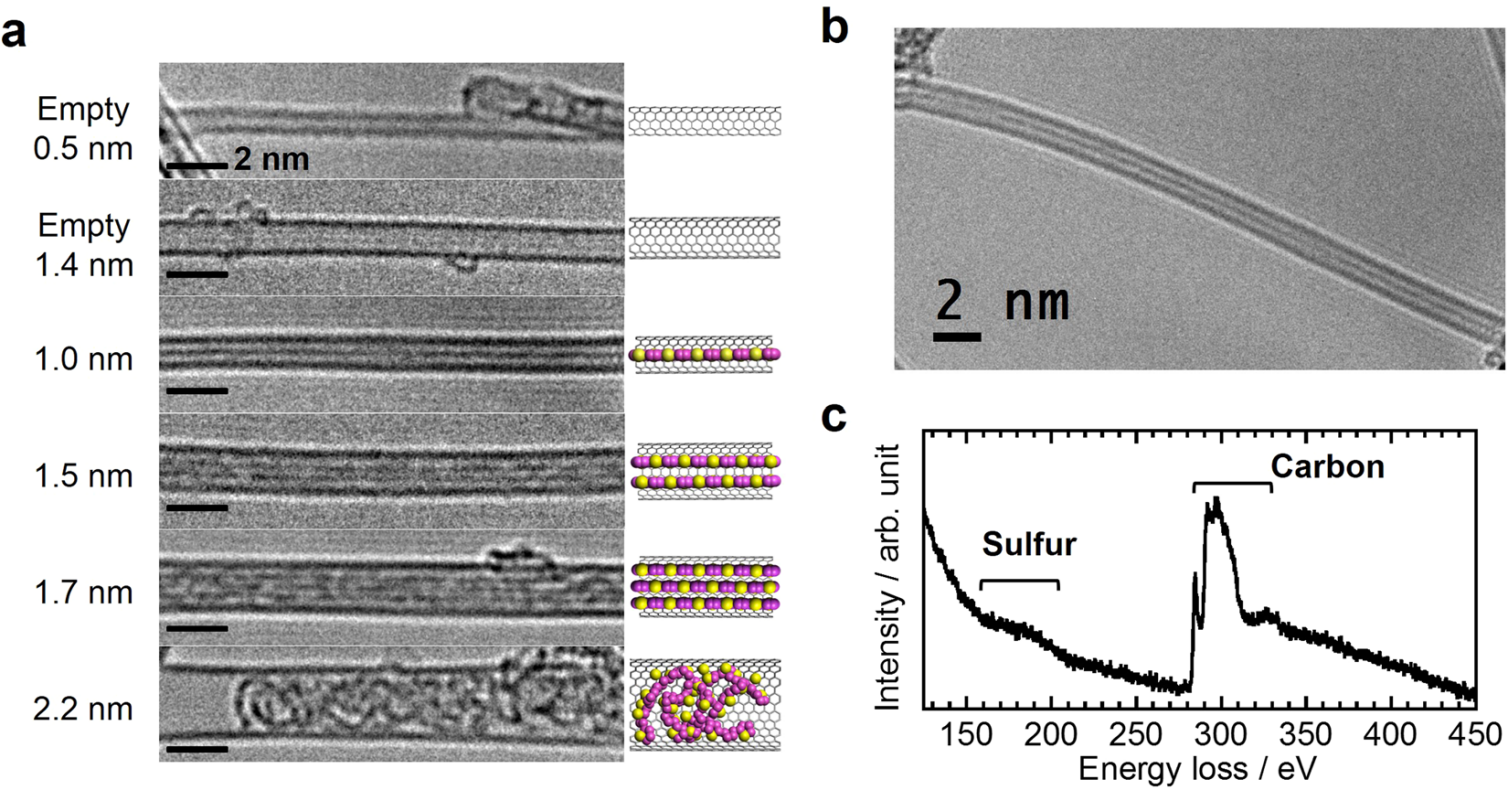
Encapsulation of thiophene oligomers in CNTs having various diameters. (**a**) HR-TEM images of 0.5 and 1.4 nm diameter CNTs before 6 T encapsulation, and 1.0, 1.5, 1.7 and 2.2 nm diameter CNTs after encapsulation, along with structural models. (**b**) HR-TEM image of a CNT exhibiting a linear contrast with a length of more than 20 nm. All encapsulated samples were thermally annealed at 400 °C. (**c**) EELS spectrum of a CNT with encapsulated 6 T.

We initially investigated the effect of the nanotube diameter on the filling yields and structures of the encapsulated materials. Figure 2a show HR-TEM images of CNTs with diameters from 0.8 to 2.0 nm before and after encapsulation of 6 T at 400 °C. In comparison to an empty nanotube, a linear contrast is observed in the CNT interiors following the encapsulation process for tubes with diameters in the range of 1.0 to 1.7 nm. The contrast lines reach a maximum length of at least 20 nm (Fig. 2b), and the number of such lines varies from one to three depending on the tube diameter. These lines are parallel to the tube axis, with the distance between the CNT sidewall and the nearest inner line being approximately 0.4 nm. This distance agrees with a previously reported value for the distance between the sidewall and 6 T^22^. The presence of sulfur in CNTs is confirmed by using electron energy loss spectroscopy (EELS) (Figs 2c and S1). It therefore appears that the 6 T is either stacked or polymerized to give polythiophenes within the CNTs, as illustrated in Fig. 1.

It should be noted that this type of linear contrast has never been observed for tubes with a 0.5 nm diameter, due to the minimal internal space. In contrast, tubes greater than 2 nm in diameter have been shown to contain randomly entangled, string-like material. These observations suggest that a suitable amount of internal space is essential for the formation of one-dimensional linear structures of thiophene polymers. Based on the above, this work focused on the nanotubes exhibiting polymer-like linear contrast.

### Effect of encapsulation temperature

**Figure 3:**
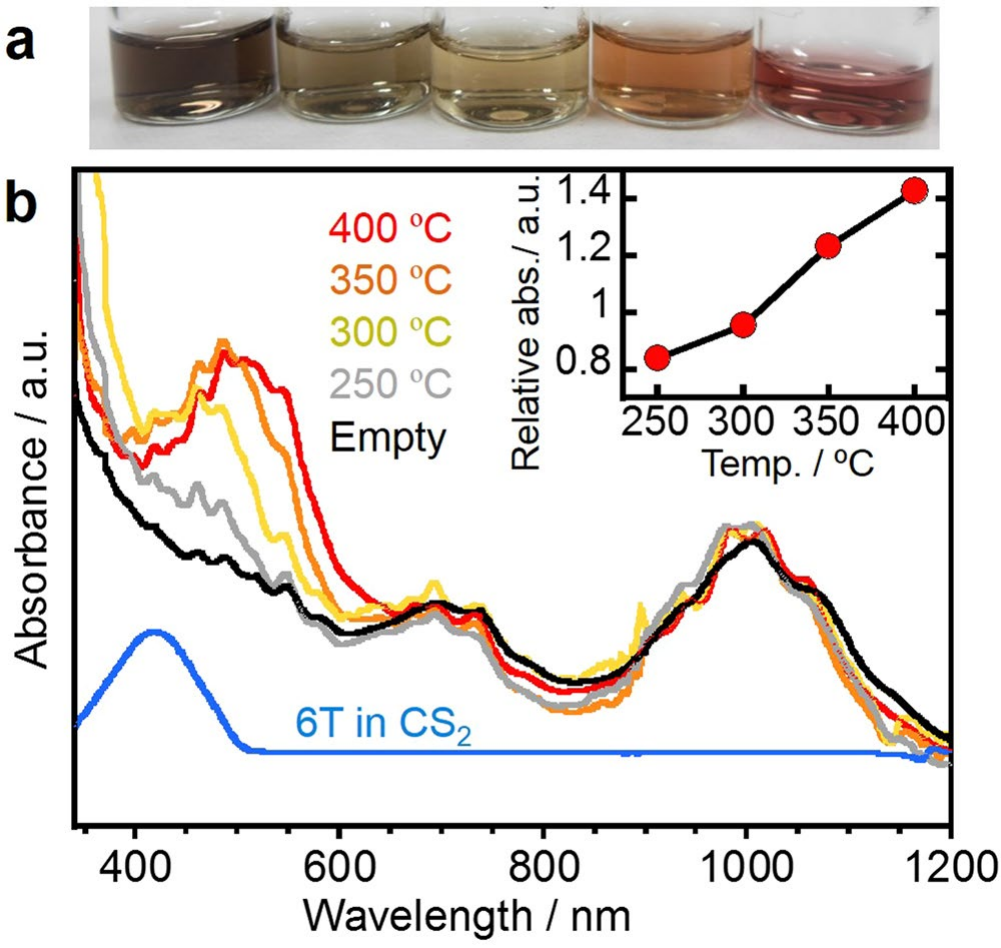
Effect of encapsulation temperature on optical absorption spectra. (**a**) Photographic images and (**b**) optical absorption spectra of CNTs dispersed in aqueous solution with a surfactant after the density gradient ultracentrifugation process. From left to right, the five samples in the images correspond to CNTs before annealing and after annealing at 250, 300, 350 and 400 °C. For a reference, absorption spectrum of a solution of 6 T in CS_2_ is shown. Inset shows the relative absorbance at 548 nm, which is defined as the ratio of absorbance at 548 nm to the S22 peak around 1000 nm, plotted as a function of annealing temperature.

To obtain further evidence for polythiophene formation, we investigated the optical responses of the samples. The formation of π-conjugated polythiophenes should extend the π-electron systems (that is, increase the effective conjugation length (ECL) of the thiophene oligomers), assuming that the products maintain the 1D translational symmetry of the thiophene units. To test this hypothesis, we acquired optical absorption spectra of samples produced by applying different annealing temperatures during the encapsulation process. For optical absorption measurement, the CNTs with encapsulated 6 T were separated from the non-encapsulated 6 T and other materials by density gradient ultracentrifugation (Fig. S2). As shown in Fig. 3, annealing at higher temperatures increased the absorbance in the vicinity of 400 to 600 nm relative to that of the S_22_ (1000 nm) and M_11_ (700 nm) absorption bands associated with the excitonic optical transitions of semiconducting and metallic CNTs having an average diameter of 1.5 nm, respectively^23^. The absorption band in the vicinity of 500 nm can be assigned to an optical transition of the encapsulated thiophenes, because a similar peak is obtained at 420 nm from a solution of 6 T in CS_2_. The increased intensity of this peak indicates a high degree of encapsulation during vapor phase process with 6 T, as shown in the inset of Fig. 3b. Importantly, the absorption peaks in the range 400 to 600 nm exhibit a red shift at higher annealing temperatures. This can be explained by a narrowing of the HOMO−LUMO gap as the ECL of the thiophene oligomers is increased^24,25^.

The same trend was also confirmed by HR-TEM observations (Fig. S3a–d). The long, linear contrast evident in Fig. 1 is associated with the high yield (>90%) obtained when the encapsulation was conducted at temperatures above 350 °C. Employing either 250 or 300 °C for the encapsulation generated relatively short (approximately 5 nm) linear chains in the nanotubes, in conjunction with significant empty space. These differences in structure and filling yield were also assessed by acquiring Raman spectra of the samples (Fig. S3e). The annealing temperature was found to affect both the peak intensity and the shape of the thiophene-derived Raman modes in the range 1400 to 1550 cm^−1^ ^26^. Furthermore, the shape of the spectra was also sensitive to the excitation wavelength (Fig. S4). This resonance effect is similar to the results obtained with coronene oligomers in our previous study^6,8^, and can be attributed to the presence of various oligomers in CNTs having different ECLs. It is also noteworthy that very similar products were obtained from the encapsulation of 4 T as well as 6 T (Fig. S5). Both HR-TEM images and Raman spectra demonstrate the polymerization of both 4 T and 6 T via dehydrogenation upon thermal annealing. These results indicate that the thermal treatment within nanotubes can induce the polymerization which involves C-H bond dissociation as well as recombination of radicals to form C-C bonds between the thiophenes as shown in Fig. 1.

### Raman imaging of encapsulated CNTs

The strong absorption of these samples prompted us to assess the potential application of the present hybrid materials to optical imaging and optoelectronic applications, especially in Raman imaging. Typically, π-conjugated polymers exhibit intense PL in the visible region. However, in the case of polymers within CNTs, this PL is oftentimes quenched through highly efficient energy transfer to the CNTs, as has been reported for carotenoids encapsulated in CNTs^27^. As a result, the Raman signal dominates the optical spectra during excitation, as seen in Figs S3–5. Raman imaging is one of the most powerful tools for high-throughput measurement of nanotube location, density and orientation on various substrates and even in living cells^28–31^. In particular, the encapsulated dye molecules are an efficient maker for individual nanotubes because imaging can be performed with single-wavelength laser excitation^28^. In contrast, complete Raman imaging of nanotubes requires laser excitation at multiple wavelengths because the resonance energies for CNTs will vary significantly depending on the chirality of the nanotubes^23,32^.

**Figure 4:**
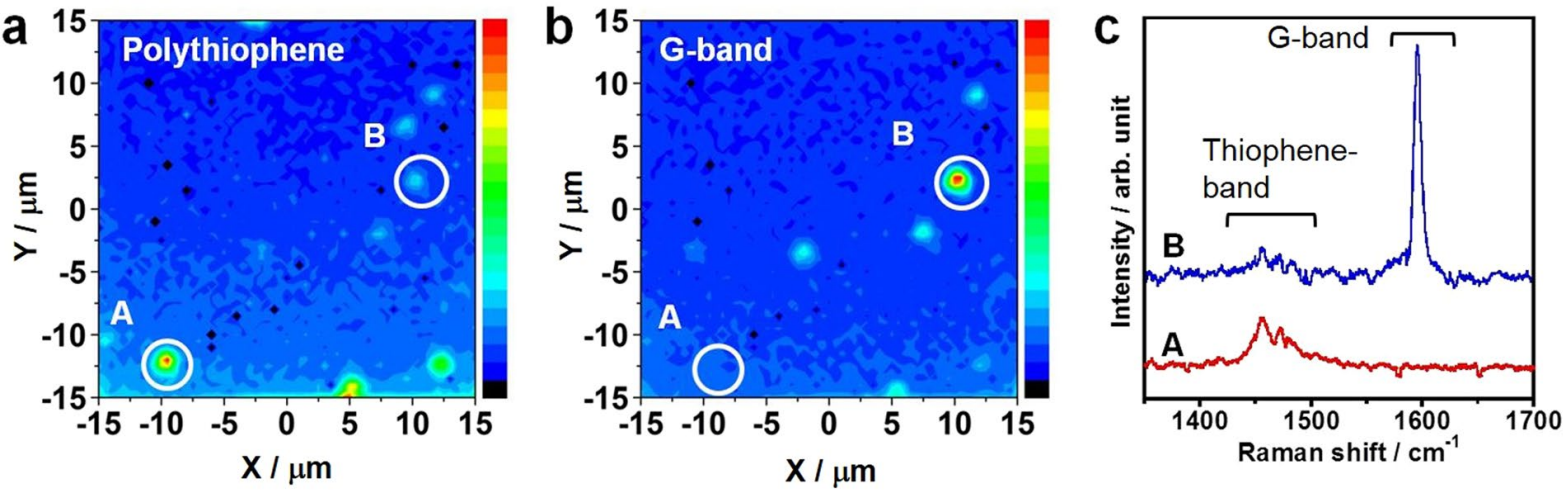
Thiophene oligomers/polymers as efficient Raman imaging markers for CNTs. Raman imaging using the (**a**) thiophene band intensity and (**b**) G-band intensity of CNTs. (**c**) Raman spectra at the points indicated by circles A and B in a and b. These data were acquired at an excitation laser wavelength of 488 nm.

To enhance the imaging performance, the present polymers have an advantage due to their strong absorption as shown in Fig. 3 as compared with the dye molecules reported in the previous study^28^. For a demonstration, individual nanotubes were deposited on a SiO_2_/Si substrate and confocal Raman imaging was performed using a 488 nm excitation laser. The resulting Raman images obtained using the thiophene band exhibit more distinct points in the same region (Fig. 4a) compared with the G-band intensity image of CNTs (Fig. 4b). In some locations, such as point A in Fig. 4a,b, only the thiophene peaks can be observed because the CNTs are not resonant at the excitation wavelength applied (Fig. 4c). These results indicate that the present hybrid system shows significant potential as a marker for the versatile imaging of CNTs in electronic and biological applications.

We also propose that Raman imaging of thiophenes could be used for the rapid identification of nanotubes between the source and drain electrodes of a nanotube-based field-effect transistor (FET, Fig. S6). The transfer curve obtained from this device shows typical p-type behavior, as has been observed previously for similar back-gate, single-CNT FETs^33^. These results demonstrate that the encapsulation of these oligomers/polymer has only a minimal effect on the electronic properties of the CNTs.

### Electrochemical doping

**Figure 5:**
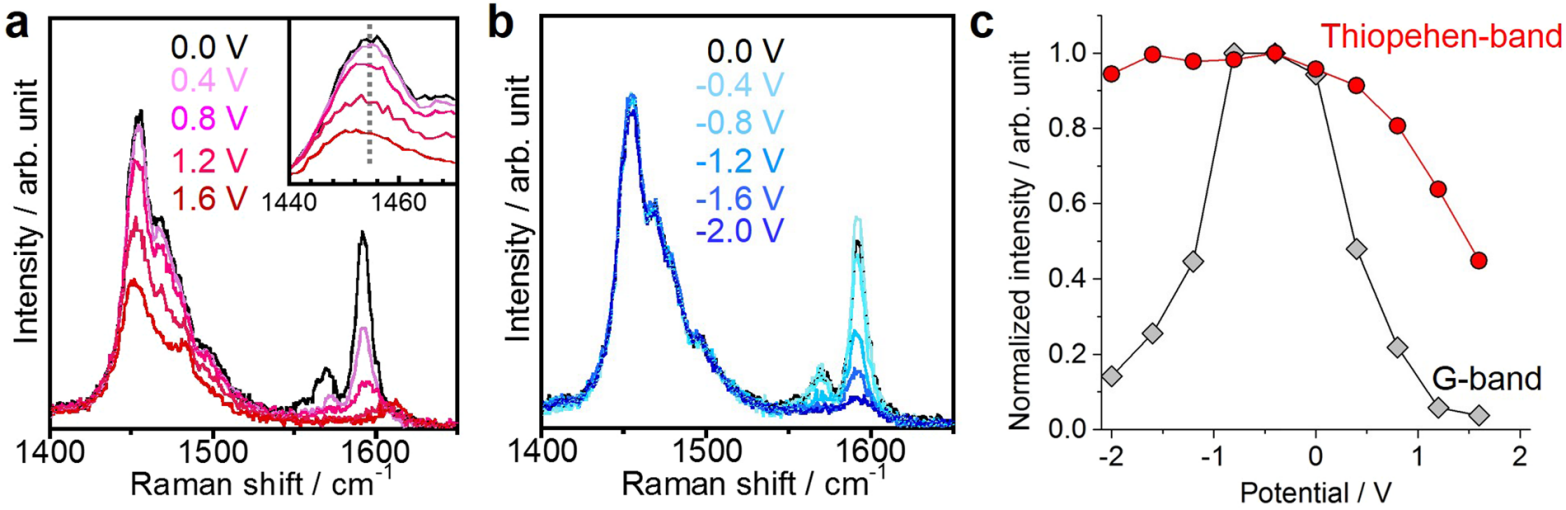
Modulation of optical spectra of thiophene oligomers/polymers in CNTs by electrochemical doping. Raman spectra acquired at varying applied (**a**) positive and (**b**) negative voltages. Inset in a shows the enlarged spectra of thiophene band. (**c**) Normalized intensities for thiophene Raman modes and nanotube G-bands as functions of the applied voltage.

In a previous study of fullerene-encapsulated nanotubes, potassium intercalation was used for the doping-induce formation of metallic polymers of C_60_^13^. The carrier doping is, therefore, also an interesting challenge to realize a novel metallic conjugated polymers inside nanotubes. For the carrier control, this work evaluated the electrochemical doping of the inner materials, as reported in a previous paper^34^. The doping level was monitored by Raman and optical absorption spectra. As shown in Fig. 5a, upon applying a positive voltage, the peak intensity for the thiophene Raman mode is reduced, and the Raman mode around 1450 cm^−1^ shifts from 1455 to 1452 cm^−1^. The optical absorption spectra also exhibit decreases in the intensities of the S_11_ and S_22_ peaks of the semiconducting CNTs (Fig. S7). This type of modulation is not observed when applying a negative voltage (Fig. 5b). The variations in the normalized intensities of each Raman band are summarized in Fig. 5c. It should be noted that these changes are reversible, excluding the possibility of electrochemical degradation of samples. These spectral changes, therefore, provide an evidence of the hole doping into the encapsulated thiophene chains, indicating the potential for forming one-dimensional conductive wires.

In summary, we have demonstrated the extension of the π-electron systems of thiophene oligomers/polymers in the interiors of CNTs. This extension was obtained via thermal annealing and was monitored by optical absorption, Raman spectroscopy and HR-TEM observations. This work also determined that such polymers can be used as efficient markers for individual CNTs during Raman imaging in conjunction with single wavelength laser excitation. Furthermore, the stable injection of holes into the encapsulated polymers was achieved by electrochemical doping, showing the possibility of forming π-electron-based conductive wires and highly stable electrochromic devices. The present results, therefore, suggest a basic strategy for the creation of novel π-conjugated polymers with exceptional functional properties and various applications.

## Methods

Sample preparation. The four CNT samples used for the TEM observations in the present study were: (1) HiPco (raw material produced by the HiPco process Carbon Nanotechnologies, Inc.) for 0.5 and 1.0 nm diameter CNTs, (2) Arc (purified material produced by arc discharge, Type-SO, Meijo Nano Carbon, Inc.) for 1.3~1.5 nm diameter CNTs, (3) Laser (material produced by laser ablation^35^ and purified by acid treatment^36^ in our laboratory) for 1.3~1.5 nm diameter CNTs, and (4) DIPS (raw material produced by direct injection pyrolytic synthesis (DIPS), Nikkiso Co., Ltd.) for 1.7 and 2.2 nm diameter CNTs. Arc samples were used for optical absorption, Raman imaging, and electrochemical doping experiments. First, CNT samples were dispersed in methanol by sonication, and then were collected on membrane filters (Omnipore menbrane with a 1 micron filter pore size, Merck Millipore) through vacuum filtration to form CNT papers. All samples were annealed between 350 and 550 °C in air for 10 to 30 min to open the nanotube caps. The required annealing time and temperature varied with the sample, and the annealing process was stopped when the sample mass had been reduced by approximately 20%.

Encapsulation was performed by placing the CNTs inside an H-shaped Pyrex glass tube, followed by degassing via heating with a burner under vacuum for 5 to 10 min. A 5 to 10 mg quantity of 6 T (Sigma-Aldrich) was then added to the ampule and the vessel was sealed under vacuum (10^−4^ Pa). It is noted that the CNT papers and 6 T powder were loaded into the different compartments of glass tube to degass them separately by a gas burner under vacuum, as shown in Fig. S8. The encapsulation of 4 T was conducted in the same manner. The glass tube was subsequently annealed at 250 to 400 °C for 24 h in a furnace. After annealing, the samples were washed ten times with CS_2_ to remove residual thiophene attached to the outer surfaces. During the washing, the CNT papers were stirred in CS_2_ solution with a magnetic stirrer. The samples were subsequently dispersed in 1% weight per volume (w/v) aqueous sodium deoxycholate (DOC) using an ultrasonic homogenizer (Sonifire 450D, Branson, power density of 20 W/cm^2^) for 12 h. During the sonication, the solution was immersed in a cold water bath to prevent heating. The solution was then separated using density gradient ultracentrifugation (DGU) at 52 krpm for 12 h using a swing bucket S52ST rotor (Hitachi Koki). Prior to the DGU process, the sample dispersion (2 mL, 30% w/v iodixanol, purchased as OptiPrep from Daiichi Pure Chemicals Co., 1% w/v DOC) was prepared in stepped gradients made using layers with the following iodixanol concentrations and volumes: 20% (2 mL) and 15% (1 mL) as shown in Fig. S1. The surfactant concentration in this gradient was fixed at 1% w/v DOC. Following centrifugation, each solution was fractionated using a pipet as shown in Fig. S1 and then used for optical absorption measurements.

### Characterization

HR-TEM observations were performed using a JEOL JEM-2100F, operating at an 80 keV accelerating voltage at room temperature. HR-TEM and EELS measurements were also performed using JEOL JEM-ARM200F with a CEOS probe and image aberration correctors using an acceleration voltage of 80 keV at room temperature. During sample preparation, methanol was added to the dispersed DOC solution and the solution was subsequently dropped onto a copper grid coated with a thin carbon film. The grids were heated under vacuum (10^−4^ Pa) at 250 °C to remove excess methanol and were then observed by HR-TEM.

The optical absorption spectra were acquired with a UV-vis-NIR spectrophotometer (JASCO V-570). Raman spectra were obtained using a backscattering geometry with a single monochromator (HR-800, Horiba Jobin Yvon) in conjunction with a charge-coupled device detector and an edge filter. The samples were excited by an argon ion laser emitting at 488 nm (2.54 eV) and a helium-neon laser emitting at 633 m (1.96 eV). Electrochemical doping was conducted to introduce charges into CNTs encapsulating 6 T, using a method reported previously^34^. Briefly, a CNT thin film was prepared on a membrane filter by vacuum filtration, and subsequently transferred to a quartz substrate with deposited Ti/Pt as electrodes. The substrate was then dipped into an ionic liquid (N,N,N-trimethyl-N-propylammonium-bis(trifluoromethanesulfonyl) imide, TMPA-TFSI).

## Electronic supplementary material

Supporting Information

## References

[1] Bandow S, Takizawa M, Hirahara K, Yudasaka M, Iijima S (2001). Raman scattering study of double-wall carbon nanotubes derived from the chains of fullerenes in single-wall carbon nanotubes. Chem. Phys. Lett..

[2] Wang Z (2010). Mixed low-dimensional nanomaterial: 2D ultranarrow MoS2 inorganic nanoribbons encapsulated in quasi-1D carbon nanotubes. J. Am. Chem. Soc..

[3] Kitaura R, Imazu N, Kobayashi K, Shinohara H (2008). Fabrication of metal nanowires in carbon nanotubes via versatile nano-template reaction. Nano Lett..

[4] Chuvilin A (2011). Self-assembly of a sulphur-terminated graphene nanoribbon within a single-walled carbon nanotube. Nat. Mater..

[5] Lim HE (2013). Growth of carbon nanotubes via twisted graphene nanoribbons. Nat. Commun..

[6] Fujihara M (2012). Dimerization-initiated preferential formation of coronene-based graphene nanoribbons in carbon nanotubes. J. Phys. Chem. C.

[7] Talyzin AV (2011). Synthesis of graphene nanoribbons encapsulated in single-walled carbon nanotubes. Nano Lett..

[8] Lim HE (2015). Fabrication and optical probing of highly extended, ultrathin graphene nanoribbons in carbon nanotubes. ACS Nano.

[9] Chernov AI (2013). Optical properties of graphene nanoribbons encapsulated in single-walled carbon nanotubes. ACS Nano.

[10] Chamberlain TW (2012). Size, structure, and helical twist of graphene nanoribbons controlled by confinement in carbon nanotubes. ACS Nano.

[11] Botka B (2014). Interactions and chemical transformations of coronene inside and outside carbon nanotubes. Small.

[12] Britz, D. A., Khlobystov, A. N., Porfyrakis, K., Ardavan, A. & Briggs, G. A. D. Chemical reactions inside single-walled carbon nano test-tubes. *Chem. Commun*., 37–39 (2005). 10.1039/b414247k 15614364

[13] Pichler T, Kuzmany H, Kataura H, Achiba Y (2001). Metallic polymers of C 60 inside single-walled carbon nanotubes. Phys. Rev. Lett..

[14] Nakanishi R (2013). Thin single-wall BN-nanotubes formed inside carbon nanotubes. Sci. Rep..

[15] Zhang J (2013). Evidence of diamond nanowires formed inside carbon nanotubes from diamantane dicarboxylic acid. Angew. Chem. Int. Ed..

[16] Nakanishi Y (2015). Template synthesis of linear‐chain nanodiamonds inside carbon nanotubes from bridgehead‐halogenated diamantane precursors. Angew. Chem. Int. Ed..

[17] Zhao C, Kitaura R, Hara H, Irle S, Shinohara H (2011). Growth of linear carbon chains inside thin double-wall carbon nanotubes. J. Phys. Chem. C.

[18] Shi L (2016). Confined linear carbon chains as a route to bulk carbyne. Nat. Mater..

[19] Miyata Y (2010). Solution-phase extraction of ultrathin inner shells from double-wall carbon nanotubes. ACS Nano.

[20] Facchetti A (2010). π-Conjugated polymers for organic electronics and photovoltaic cell applications. Chem. Mater..

[21] Moliton A, Hiorns RC (2004). Review of electronic and optical properties of semiconducting π-conjugated polymers: applications in optoelectronics. Polym. Int..

[22] Loi MA (2010). Encapsulation of Conjugated Oligomers in Single‐Walled Carbon Nanotubes: Towards Nanohybrids for Photonic Devices. Adv. Mater..

[23] Kataura H (1999). Optical properties of single-wall carbon nanotubes. Synth. Met..

[24] Zade SS, Bendikov M (2006). From oligomers to polymer: Convergence in the HOMO− LUMO gaps of conjugated oligomers. Org. Lett..

[25] Becker RS, S de Melo J, Macanita AL, Elisei F (1996). Comprehensive evaluation of the absorption, photophysical, energy transfer, structural, and theoretical properties of α-oligothiophenes with one to seven rings. J. Phys. Chem..

[26] Chen Fe, Shi G, Zhang J, Fu M (2003). Raman spectroscopic studies on the structural changes of electrosynthesized polythiophene films during the heating and cooling processes. Thin Solid Films.

[27] Yanagi K (2006). Light-harvesting function of β-carotene inside carbon nanotubes. Phys. Rev. B.

[28] Gaufrès E (2014). Giant Raman scattering from J-aggregated dyes inside carbon nanotubes for multispectral imaging. Nat. Photonics.

[29] Havener RW (2011). High-throughput graphene imaging on arbitrary substrates with widefield Raman spectroscopy. ACS Nano.

[30] Kang JW, Nguyen FT, Lue N, Dasari RR, Heller DA (2012). Measuring uptake dynamics of multiple identifiable carbon nanotube species via high-speed confocal Raman imaging of live cells. Nano Lett..

[31] Havener RW (2012). High-Throughput Graphene Imaging on Arbitrary Substrates with Widefield Raman Spectroscopy. ACS Nano.

[32] Weisman RB, Bachilo SM (2003). Dependence of optical transition energies on structure for single-walled carbon nanotubes in aqueous suspension: an empirical Kataura plot. Nano Lett..

[33] Kim W (2003). Hysteresis caused by water molecules in carbon nanotube field-effect transistors. Nano Lett..

[34] Yanagi K, Moriya R, Cuong NT, Otani M, Okada S (2013). Charge manipulation in molecules encapsulated inside single-wall carbon nanotubes. Phys. Rev. Lett..

[35] Kataura H (2000). Diameter control of single-walled carbon nanotubes. Carbon.

[36] Miyata Y, Yanagi K, Maniwa Y, Kataura H (2008). Optical evaluation of the metal-to-semiconductor ratio of single-wall carbon nanotubes. J. Phys. Chem. C.

