# Supplementary material for: Extended-conjugation π-electron systems in carbon nanotubes
Source: Sci Rep. 2018 May 25;8:8098. doi: 10.1038/s41598-018-26379-4 (PMC5970159; doi:10.1038/s41598-018-26379-4)
Supplement: Supplementary file 1 — Supporting Information [file 41598_2018_26379_MOESM1_ESM.docx]

Supporting Information

**Extended-conjugation π-electron systems in carbon nanotubes**

Kenshi Miyaura^1^, Yasumitsu Miyata^2,3,*^, Boanerges Thendie^1^, Kazuhiro Yanagi^2^, Ryo Kitaura^1^, Yuta Yamamoto^4^, Shigeo Arai^4^, Hiromichi Kataura^5^, Hisanori Shinohara^1,*^

*^1^Department of Chemistry & Institute for Advanced Research, Nagoya University,*

*Nagoya 464-8602, Japan*

*^2^Department of Physics, Tokyo Metropolitan University, Hachioji 192-0397, Japan*

*^3^JST, PRESTO, Kawaguchi, Saitama 332-0012, Japan*

*^4^High Voltage Electron Microscope Laboratory, Ecotopia Science Institute, Nagoya University, Nagoya 464-8602, Japan*

*^5^Nanomaterials Research Institute, National Institute of Advanced Industrial Science and Technology (AIST), Tsukuba, Ibaraki 305-8565, Japan*


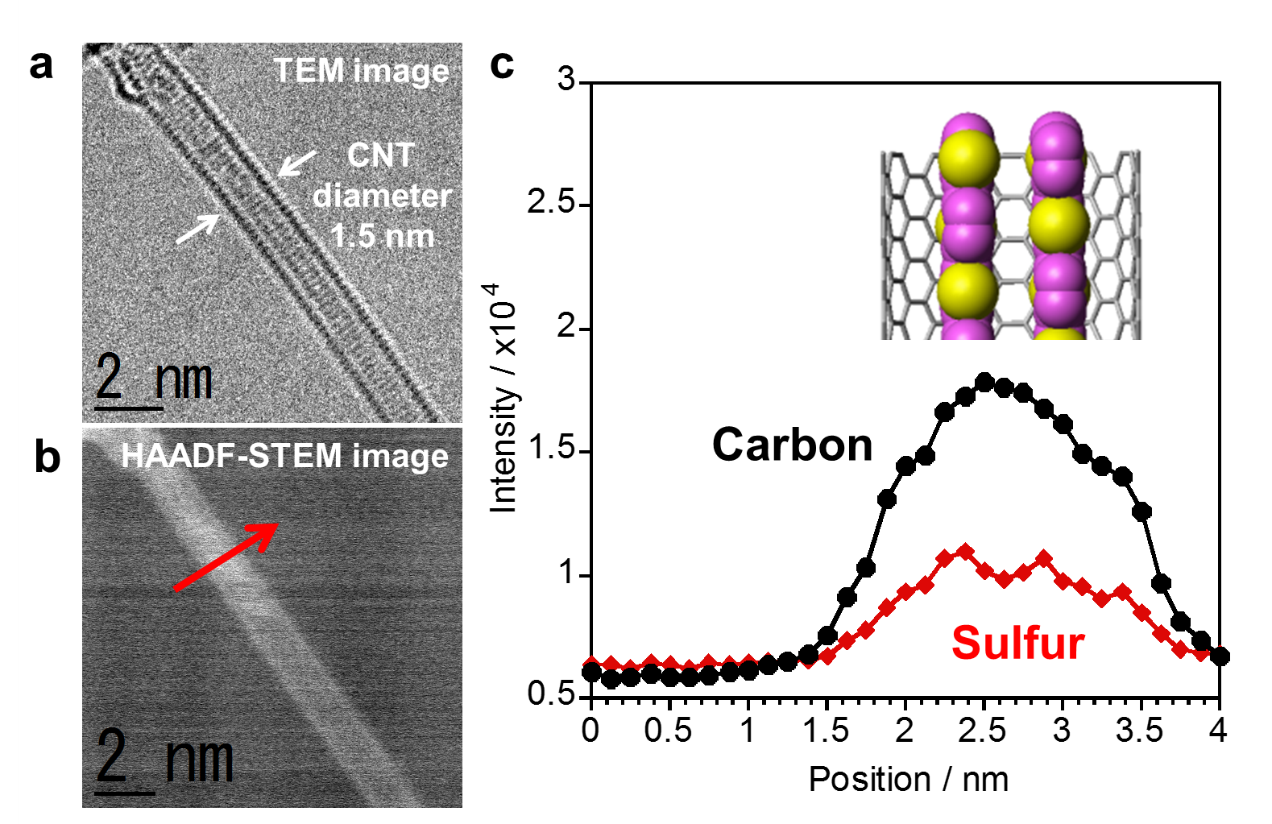


**Figure S1**. (a) Bright-field HR-TEM and (b) high-angle annular dark-field scanning-transmission electron microscope (HAADF-STEM) images of a CNT with encapsulated 6T. (c) Intensity profiles of sulfur (red) and carbon (black) peaks in EELS spectra recorded along the red arrow in b. The presence of two peaks of sulfur profile inside a CNT agrees with two linear contrasts in a, as illustrated in structure mode.


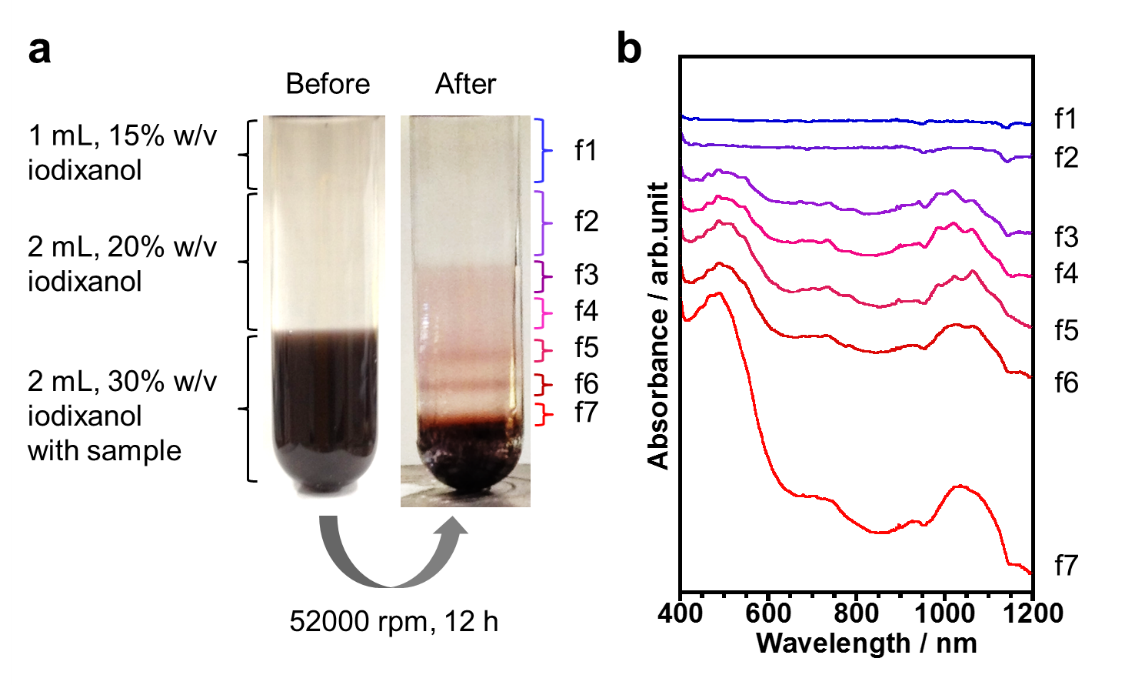


**Figure S2.** (a) Photographic images acquired before and after density gradient ultracentrifugation. (b) Optical absorbance spectrum of each fraction. Fraction 3 (f3) was used for characterizations.


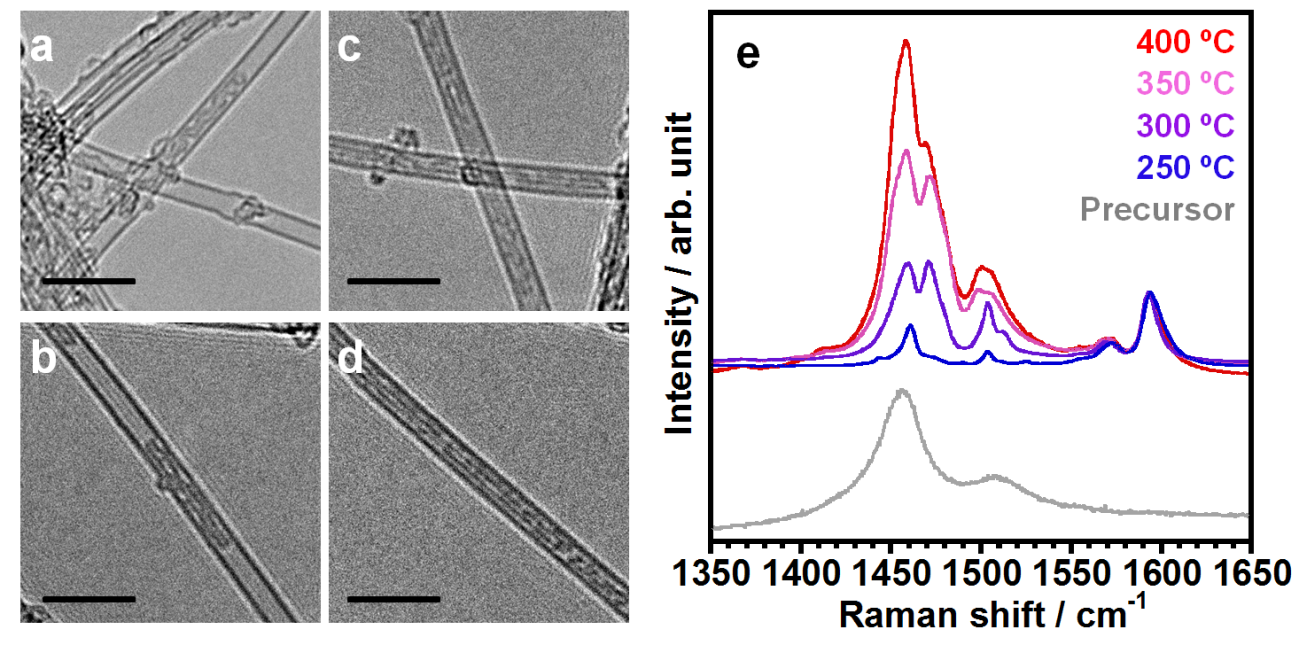


**Figure S3**. HR-TEM images of encapsulated CNTs obtained at different encapsulation temperatures: (a) 250, (b) 300, (c) 350 and (d) 400 ºC. Scale bars are 5 nm. (e) Raman spectra of thiophene precursor (6T) and CNTs containing encapsulated 6T following application of various annealing temperatures. The spectra were acquired with a 488 nm excitation laser.


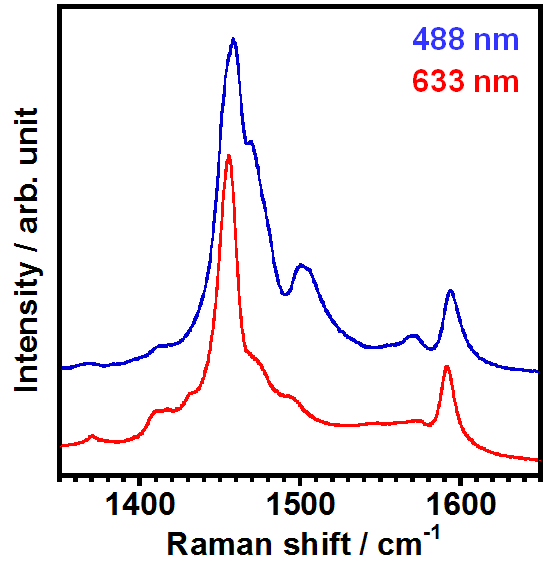


**Figure S4.** Raman spectra of CNTs with encapsulated 6T excited at two laser wavelengths: 488 nm (blue) and 633 nm (red).


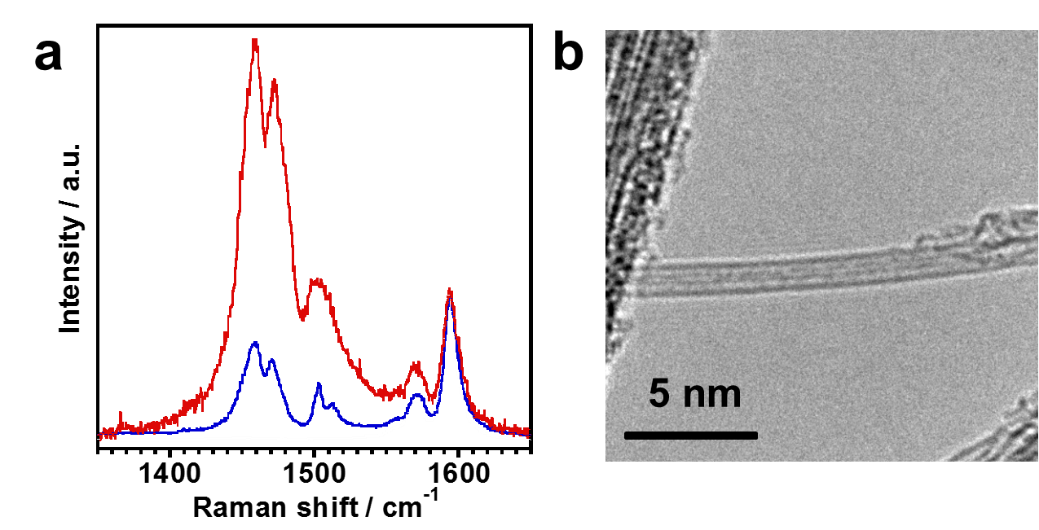


**Figure S5.** (a) Raman spectra of CNTs after encapsulation of 4T (blue) and 6T (red) at 350 ºC. The spectra were acquired with a 488 nm excitation laser. (b) HR-TEM images of CNTs with encapsulated 4T after processing at 350 ºC.


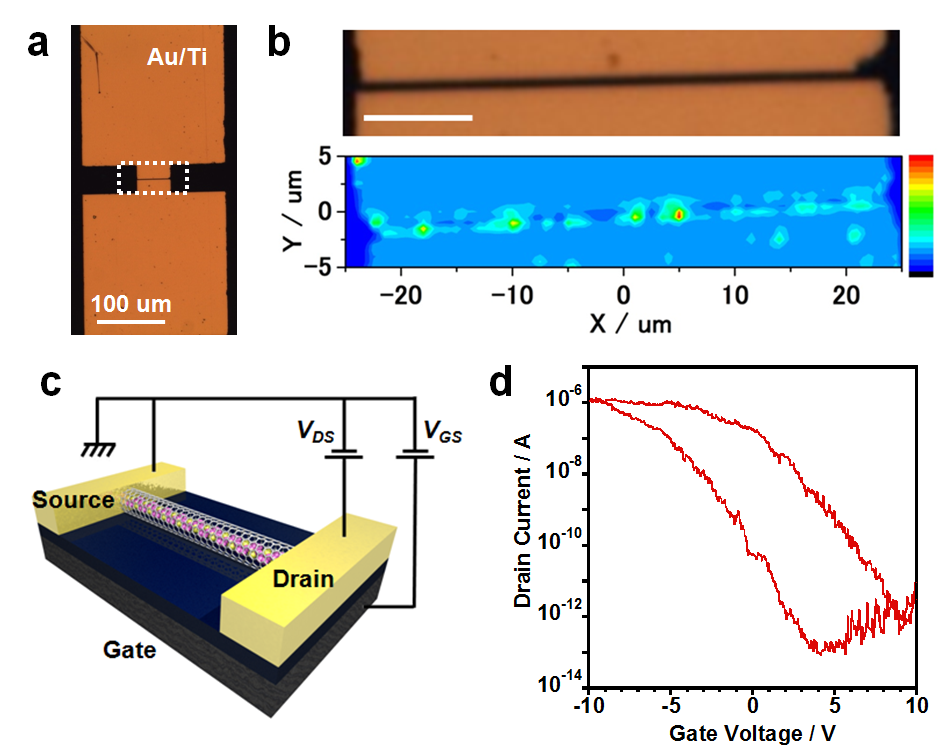


**Figure S6.** (a) Optical microscopy image of source and drain electrodes (5 nm Ti/ 20 nm Au). (b) Enlarged optical microscopy image and Raman image of thiophene band in area indicated by white dots in a. (c) Schematic illustration and (d) transfer curve of present CNT-based FET. The channel width and length in this device were 50 μm and 500 nm, respectively.


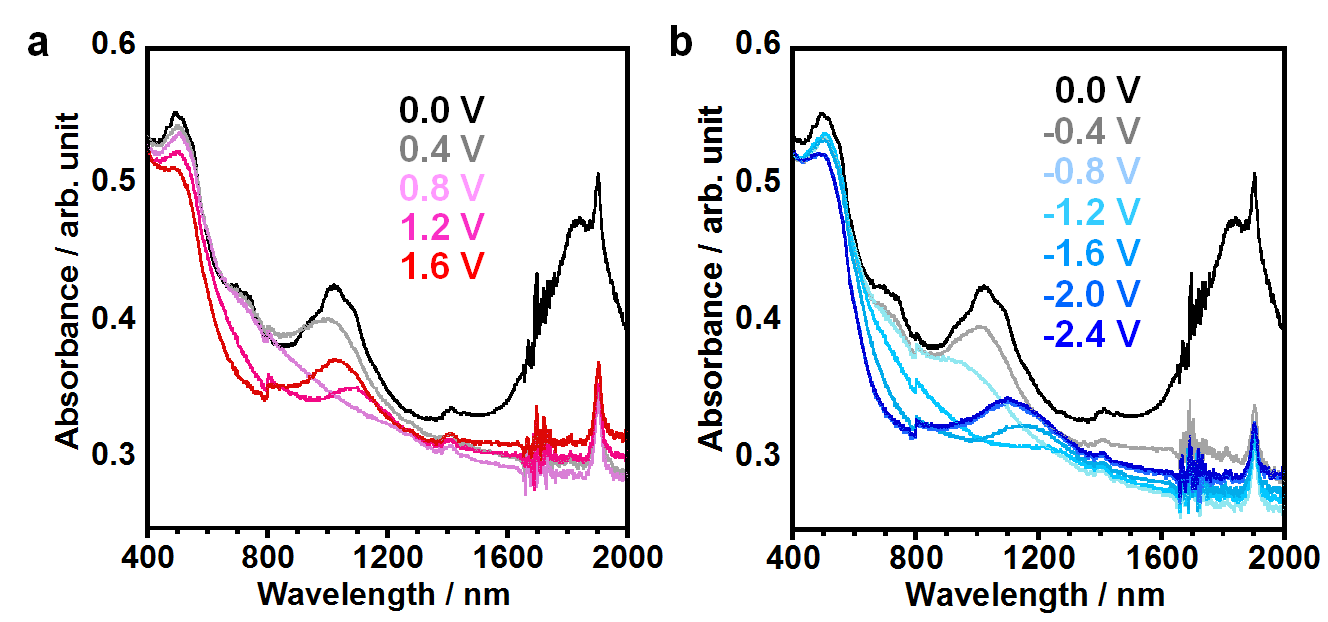


**Figure S7**. Optical absorption spectra of CNTs with encapsulated 6T at different stages of electrochemical doping during (a) hole (0 to +1.6 V vs. Ag/Ag+) and (b) electron (0 to −2.4 V shift vs. Ag/Ag+) injections.


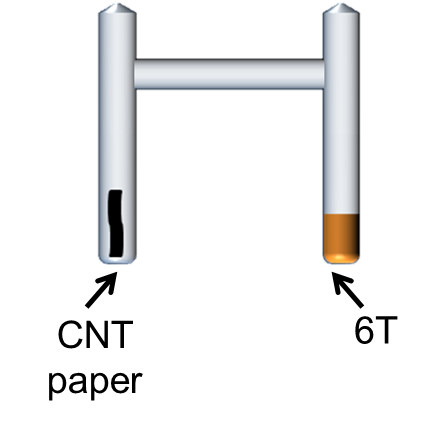


**Figure S8.** Illustration of an H-shaped Pyrex glass tube used for the encapsulation.
